# Mitochondrial complex II is a source of the reserve respiratory capacity that is regulated by metabolic sensors and promotes cell survival

**DOI:** 10.1038/cddis.2015.202

**Published:** 2015-07-30

**Authors:** J Pfleger, M He, M Abdellatif

**Affiliations:** 1 Department of Cell Biology and Molecular Medicine, Rutgers-New Jersey Medical School, Newark, NJ 07103, USA

## Abstract

The survival of a cell depends on its ability to meet its energy requirements. We hypothesized that the mitochondrial reserve respiratory capacity (RRC) of a cell is a critical component of its bioenergetics that can be utilized during an increase in energy demand, thereby, enhancing viability. Our goal was to identify the elements that regulate and contribute to the development of RRC and its involvement in cell survival. The results show that activation of metabolic sensors, including pyruvate dehydrogenase and AMP-dependent kinase, increases cardiac myocyte RRC via a Sirt3-dependent mechanism. Notably, we identified mitochondrial complex II (cII) as a target of these metabolic sensors and the main source of RRC. Moreover, we show that RRC, via cII, correlates with enhanced cell survival after hypoxia. Thus, for the first time, we show that metabolic sensors via Sirt3 maximize the cellular RRC through activating cII, which enhances cell survival after hypoxia.

During normal/unstressed conditions, the cell runs on a fraction of its mitochondrial bioenergetics capacity, where the difference between the maximum respiratory capacity and basal respiratory capacity is referred to as the spare or reserve respiratory capacity (RRC). In the case when energy demand exceeds supply (e.g., an increase in workload or neuronal activity), the RRC has the potential to increase supply, thus, avoiding an ‘ATP crisis'. In accordance, RRC has been shown to correlate with enhanced cell survival^1^ and, conversely, reduced RRC has been associated with neuronal cell death and disease.^2^ RRC is a well-recognized phenomenon;^3, 4, 5, 6, 7, 8, 9^ however, its components or the factors that regulate it remain unknown, or, at best, minimally defined. Not surprisingly, one of the known factors that influence the extent of the RRC is substrate availability.^7^

One potential source of RRC is a regulated increase of substrate entry into the TCA cycle that is synchronized with an increase in the electron transport chain (ETC) activity. Interestingly, mammalian complex II (cII) has the unique characteristic of being a common component that links the TCA cycle and the ETC and its role in cell survival and death is well established. For example, inactivating mutations in the subunit A (SDHA) are associated with Leigh's syndrome, which is a progressive neurodegenerative disease associated with neuronal cell death.^10^ Likewise, at least one case report shows that a mutation in cII is associated with heart failure,^11^ while in *Drosophila* a mutation in Sdhb causes an increase in ROS production and early mortality.^12^ In contrast, inhibition of cII during ischemia/reperfusion attenuates ROS-induced damage.^13^ Indeed, while inhibiting cII has been shown to induce apoptosis,^14^ it is also recognized as an apoptosis sensor.^15^ One mechanism that has been described for cII-induced apoptosis involves its disassembly in the low pH environment of distressed cells that results in excessive production of ROS from the Sdha.^16, 17^ Thus, these results would suggest that a fully assembled cII is critical for cell health and survival, while the disassembled form participates in cell demise. In this report, we show that holo-cII is the source of the RRC, which increases the cells' resistant to cell death.

## Results

### The RRC is dependent on the metabolic substrate in a cell type-dependent manner

**Figure 1:**
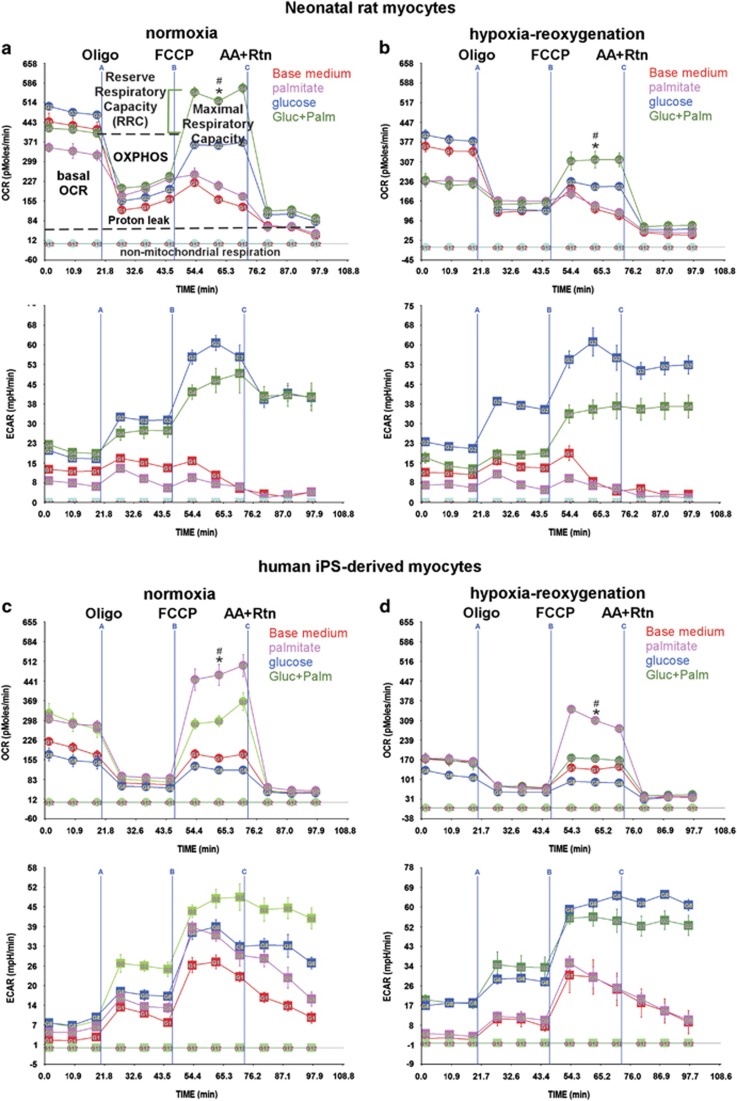
The RRC is differentially regulated by glucose and fatty acid oxidation in neonatal rat myocytes and human iPSC-C. (**a**–**d**) Neonatal rat cardiac myocytes (**a** and **b**) or human iPSC-CM (**c** and **d**) were incubated for 24 h in complete growth medium under normoxic (atmospheric O_2_) (**a** and **c**) or hypoxic (<1% O_2_) (**b** and **d**) conditions. After this period, the medium was replaced with glucose- and fatty acid-free base medium, or that containing 17.5 mM glucose, 100 *μ*M palmitate-BSA or 17.5 mM glucose plus 100 *μ*M palmitate-BSA (Gluc+Palm), as indicated, in normoxic conditions, for another 24 h. The medium was then replaced with serum-free XF medium containing the indicated substrates. The mitochondrial stress test was then performed as described in Methods and Materials, *n*=4–6, each experiment performed twice. For (**a** and **b**), error bars represent S.E.M., *P<0.05 max OCR *versus* basal OCR for Gluc+Palm at the time point indicated; ^#^*P*<0.05 max OCR for Gluc+Palm *versus* max OCR of base medium. For (**c** and **d**), error bars represent S.E.M., *P<0.05 max OCR *versus* basal OCR for palmitate at the time point indicated; ^#^*P*<0.05 max OCR for palmitate *versus* max OCR of base medium

Our first aim was to assess mitochondrial bioenergetics in live cells and the influence of metabolic substrates on oxygen consumption rates (OCRs) during normoxia *versus* post-hypoxia. In Figure 1, we measure the OCR of live neonatal cardiac myocytes maintained in atmospheric O_2_ levels. The characteristics of these cells include spontaneous contraction in culture, high mitochondrial content, and a preference for glucose as a substrate. The data show that basal OCR was the highest (510 pmoles/min/100 000 cells) with glucose in the medium (Figure 1a, upper panel), which was slightly (10–20%) dampened by palmitate-BSA (Figure 1a, upper panel). On the other hand, the presence of palmitate-BSA or amino acids (base medium with no glucose) alone resulted in 24–33% lower OCR levels *versus* control. The injection of oligomycin in the medium revealed the ATP synthesis-linked OCR (OXPHOS), which was proportional to basal OCR.

To determine the maximal respiratory capacity and, thereby, the RRC, the uncoupler p-trifluoromethoxy carbonyl cyanide phenyl hydrazone (FCCP) was injected into the medium at the time point indicated in the graph. The results show that only in the presence of both glucose and palmitate-BSA did the cells have an RRC (1.4- to 2.5-fold over basal OCR). After exposure to hypoxia (<0.1% O_2_) for 24 h (in the presence of complete medium with glucose and fetal bovine serum), the cells were allowed to recover for another 24 h with the indicated substrates. Notably, under these conditions there was no significant cell death, as determined by the quantitative PCR (qPCR) and live/dead assays (Supplementary Figures S1 and S2). As shown, the basal OCR was reduced (~30%) with all substrates, accompanied by equivalent decreases in OXPHOS, and the RRC was also downregulated (60–100%), with only partial recovery within 24 h (Figure 1b, upper panel). Thus, the data demonstrate that basal OCR and RRC have different substrate requirements, where palmitate is necessary for the development of the latter only, suggesting that these parameters constitute independent entities of the cell's bioenergetics.

To validate the development of RRC in human myocytes, we used human induced-pluripotent stem cell-derived cardiac myocytes (iPSC-CM), which are also spontaneously contracting in culture. Figure 1 shows that basal OCR levels were highest in the presence of palmitate-BSA, whether or not glucose was present (~300 pmoles/min/60 000 cells), whereas glucose or amino acid alone resulted in 33–50% lower OCR levels (Figure 1c, upper panel). In contrast to neonatal myocytes, palmitate-BSA alone produced maximum RRC during normoxia or after hypoxia (1.5- to 2.4-fold over basal OCR), which was diminished by >80% by the addition of glucose (Figures 1c and d, upper panels). Also, while monitoring OCR in the cells we are able to simultaneously monitor extracellular acidification rates (ECAR), as an indirect indicator of glycolysis (Figures 1a–d, lower panels). As seen, ECAR levels are highest in the presence of glucose and show a 200% increase in basal levels and 25% in maximum capacity after hypoxia, especially in the human myocytes (Figure 1d, lower panel). In short, fatty acids maintain maximum RRC in hiPSC-CM, which is significantly diminished by glucose, while neonatal myocytes strictly require both fatty acids and glucose to develop a reserve.

### The reserve respiratory capacity is regulated by pyruvate dehydrogenase kinases

**Figure 2:**
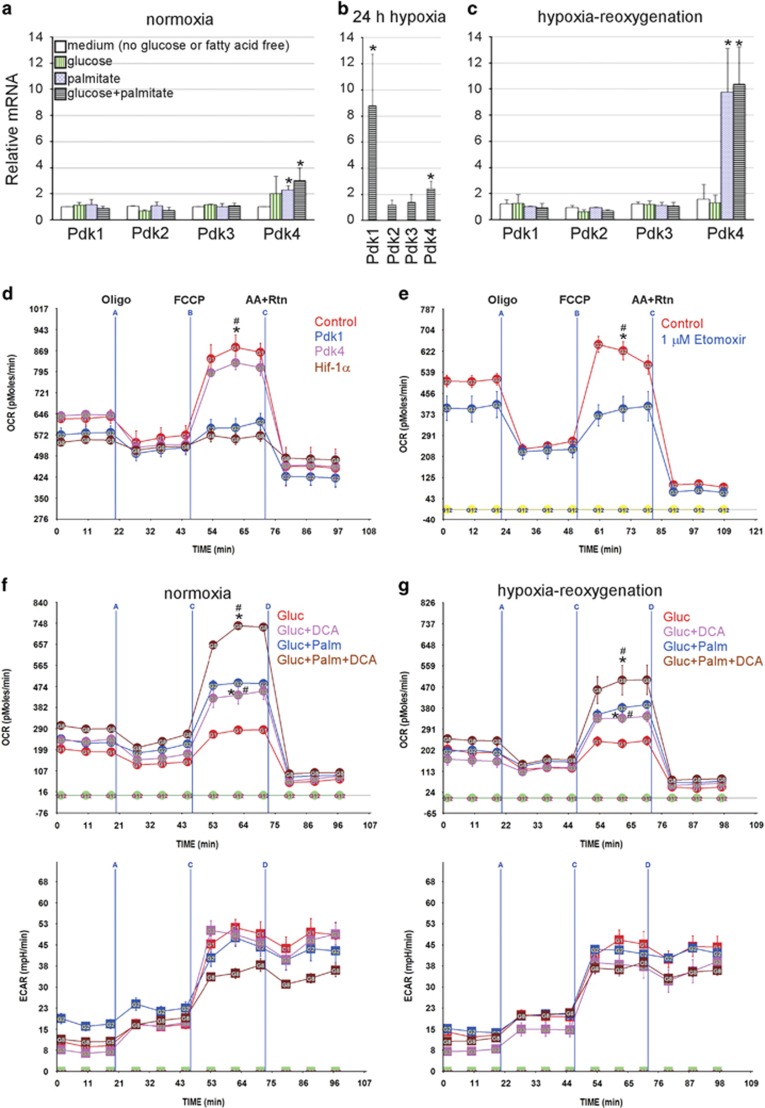
The RRC is regulated by pyruvate dehydrogenase kinases. (**a**–**c**) Neonatal rat cardiac myocytes were incubated for 24 h in complete growth medium under normoxic (atmospheric O_2_) (**a**) or hypoxic (<1% O_2_) (**b** and **c**) conditions. After this period, RNA was either immediately extracted and subjected to qPCR for the indicated genes (**b**), or the medium was replaced with one that is glucose and fatty acid free, or one containing 17.5 mM glucose, 100 *μ*M palmitate-BSA, or glucose+palmitate-BSA, as indicated, in normoxic conditions, for another 24 h (**a** and **c**). The results were averaged plotted as relative values to those from cells incubated in base medium in normoxic conditions adjusted to 1 (**a** and **c**) or control normoxia adjusted to 1 (**b**), *n*=3. Error bars represent standard error of the mean (S.E.M.), *P<0.05 *versus* control. (**d**) Neonatal cardiac myocyte was incubated for 24 h in complete growth medium and then infected with a control or adenoviruses (Ad) harboring PDK1, PDK4 or Hif-1*α*, for 24 h. The medium was then replaced with serum-free XF medium containing 17.5 mM glucose plus 100 *μ*M palmitate for 1 h. The mitochondrial stress test was then performed as described in Materials and Methods, *n*=4–6, each experiment was performed twice. Error bars represent S.E.M., *P<0.05 max OCR *versus* basal OCR for control at the time point indicated; ^#^*P*<0.05 max OCR for control *versus* max OCR of Pdk1 or Hif-1*α* treated. (**e**) Neonatal cardiac myocytes were cultured for 24 h in complete growth medium containing vehicle or 1 *μ*M etomoxir, a Cpt1 inhibitor. The medium was then replaced with serum-free XF medium containing 17.5 mM glucose plus 100 *μ*M palmitate containing vehicle or 1 *μ*M etomoxir, for 1 h. The mitochondrial stress test was then performed as described in Materials and Methods, *n*=4–6, each experiment was performed twice. Error bars represent S.E.M., *P<0.05 max OCR *versus* basal OCR for control at the time point indicated; ^#^*P*<0.05 max OCR for control *versus* max OCR for etomoxir treated, at the time point indicated (**f** and **g**). Neonatal rat cardiac myocytes were cultured in complete growth medium containing vehicle or 1 mM DCA and either remained in normoxic conditions (atmospheric O_2_) (**f**) or were exposed to hypoxia (<1% O_2_) (**g**), for 24 h. At the end of this period, the medium was changed to base medium containing 17.5 mM glucose or 17.5 mM glucose plus 100 *μ*M palmitate-BSA containing vehicle or DCA, as indicated, for an additional 24 h. The medium was then replaced with serum-free XF medium containing 17.5 mM glucose or 17.5 mM glucose plus 100 *μ*M palmitate-BSA containing vehicle or 1 mM DCA, for 1 h. The mitochondrial stress test was then performed as described in Materials and Methods, *n*=4–6, each experiment was performed three times. Error bars represent S.E.M., *P<0.05 max OCR *versus* basal OCR for DCA-treated cells, at the time point indicated; ^#^*P*<0.05 max OCR for DCA-treated *versus* max OCR for untreated, at the time point indicated

A major aspect of metabolism that is modified during hypoxia is the inhibition of glucose oxidation via hypoxia-inducible factor 1-alpha (Hif-1*α*)-mediated increase in Pdk1. Therefore, we examined the expression pattern of the Pdk isoforms during normoxia and after hypoxia/reoxygenation with different metabolic substrates, as well as, immediately following 24 h of hypoxia in complete medium. As seen in Figure 2, the different substrates did not have any impact on the expression levels of Pdks 1–3 during normoxia, with a small but significant increase in Pdk4 in the presence of palmitate (Figure 2a), while hypoxia increased the levels of Pdk1 (9-fold) and Pdk4 (2.5-fold) (Figure 2b). After 24 h of reoxygenation, Pdk1 levels returned to basal values, whereas Pdk4 continued to increase (~10-fold) in the presence of palmitate (Figure 2c). The latter could explain the lack of full metabolic recovery 24 h after hypoxia/reoxygenation.

We hypothesized that Pdks are negative regulators of the RRC. To test this, we overexpressed Pdk1, Pdk4 or Hif-1*α*, which induces the expression of Pdk1, in neonatal myocytes, in the presence of palmitate-BSA plus glucose. The results of this experiment show that both Pdk1 and Hif-1*α* completely eliminated the RRC, with minimal (~15%), but insignificant, reduction of basal OCR (Figure 2d), which adjusts via utilization of amino acids (see Figure 1a). Notably, Pdk4 had little effect on RRC under these conditions.

Since RRC requires both glucose and fatty acid, we predicted that inhibition of mitochondrial uptake of fatty acid would similarly abrogate RRC. Figure 2e proves this to be true, via inhibiting carnitine palmitoyltransferase 1 (Cpt1) with etomoxir, which resulted in complete abrogation of RRC and ~20%, but insignificant, reduction in basal OCR. These results confirm that both glucose and fatty acid function synergistically to generate an RRC in neonatal myocytes, and emphasize the fact that basal OCR and RRC have unique metabolic requirements and regulators.

To confirm the role of Pdks in regulating RRC, we treated the cells with the Pdk inhibitor dichloroacetate (DCA), which activates glucose oxidation.^18^ The results show that DCA modestly increased basal OCR (22–25%) during normoxia in cells that were supplied with glucose plus palmitate, but not glucose only (Figure 2f, upper panel). On the other hand, it more robustly increased RRC by 1.66- and 1.56-fold under those same conditions, respectively. Furthermore, hypoxia/reoxygenation reduced basal OCR and RRC of DCA-treated and untreated cells equally, however, only the DCA-treated cells, in the presence of glucose plus palmitate, retained values that are equivalent to untreated cells during normoxia, as seen in Figure 2g (upper panel). It should be noted that none of the treatments had a significant effect on ECAR (Figures 2f and g, lower panels). This suggests that the non-lethal hypoxia-induced mitochondrial dysfunction may be attributable to the sustained increase in Pdk activity and is largely preventable via its inhibition.

### The reserve respiratory capacity is regulated by AMPK

**Figure 3:**
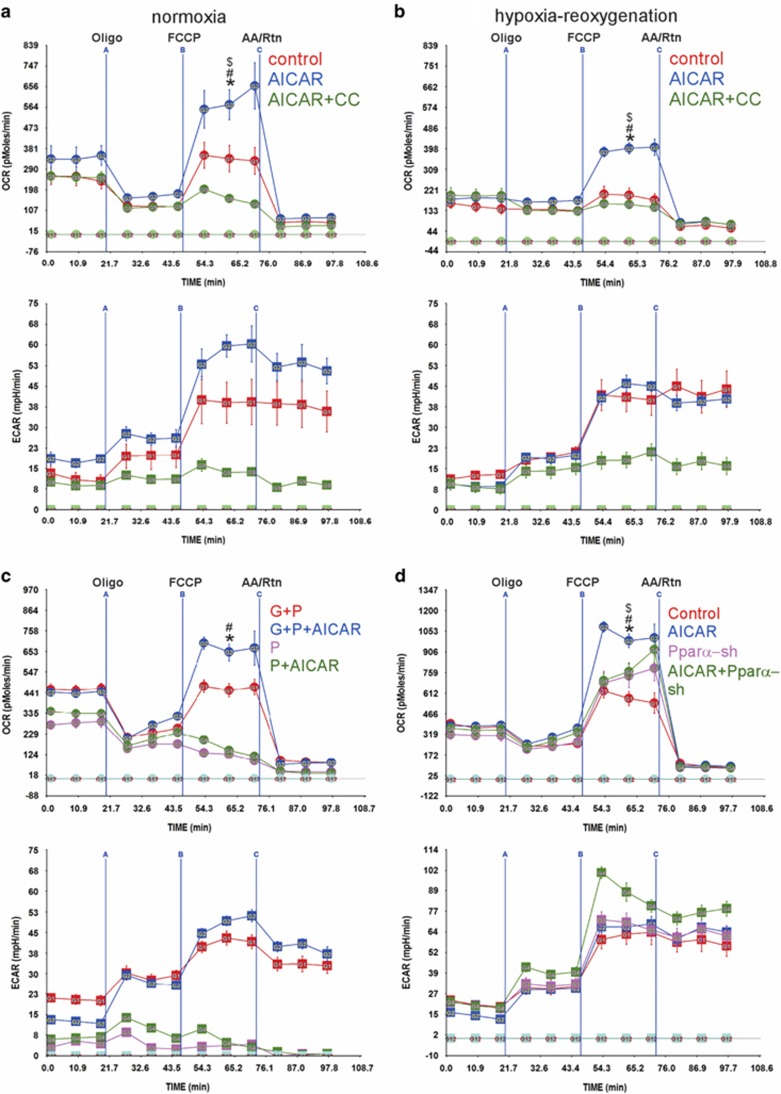

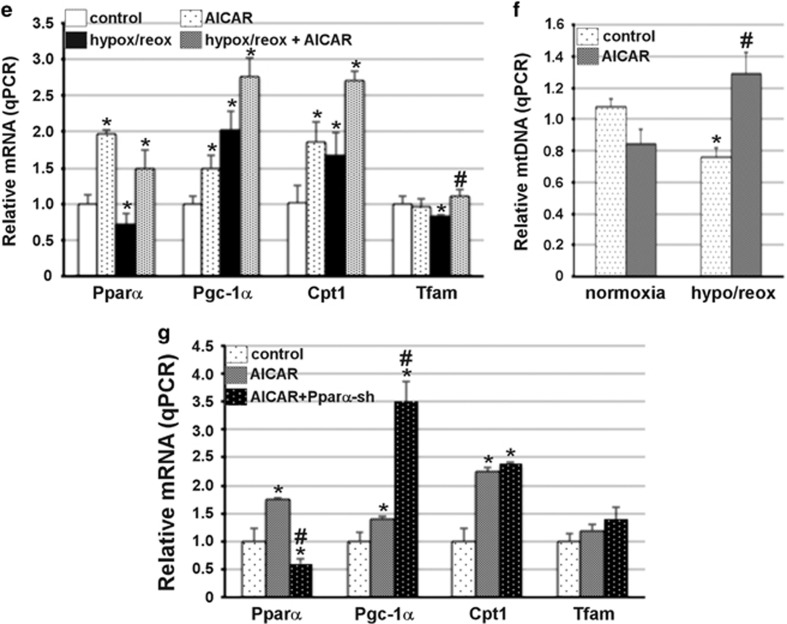
The RRC is regulated by AMPK *via* Ppar*α.* (**a** and **b**) Neonatal rat cardiac myocytes were cultured in complete growth medium containing vehicle, 500 *μ*M AICAR or 500 *μ*M AICAR plus 5 *μ*M compound C (CC) and either remained in normoxic conditions (atmospheric O_2_) (**a**) or were exposed to hypoxia (<1% O_2_), for 24 h (**b**). The medium was replaced with base medium containing 17.5 mM glucose plus 100 *μ*M palmitate-BSA containing vehicle, 500 *μ*M AICAR or 500 *μ*M AICAR plus 5 *μ*M CC, for 24 h. The medium was then replaced with serum-free XF medium containing 17.5 mM glucose plus 100 *μ*M palmitate-BSA containing vehicle, 500 *μ*M AICAR or 500 *μ*M AICAR plus 5 *μ*M CC, for 1 h. The mitochondrial stress test was then performed as described in Materials and Methods, *n*=4–6, each experiment was performed three times. Error bars represent S.E.M., *P<0.05 max OCR *versus* basal OCR for AICAR-treated cells, at the time point indicated; ^#^*P*<0.05 max OCR for AICAR-treated *versus* max OCR for control, at the time point indicated; ^#^*P*<0.05 max OCR for AICAR treated *versus* max OCR for CC treated. (**c**) Neonatal rat cardiac myocytes were treated with vehicle or 500 *μ*M AICAR in the presence of 17.5 mM glucose plus 100 *μ*M palmitate-BSA or 100 *μ*M palmitate-BSA, for 24 h. The medium was then replaced with serum-free XF medium containing 17.5 mM glucose plus 100 *μ*M palmitate-BSA or 100 *μ*M palmitate-BSA containing vehicle or 500 *μ*M AICAR, for 1 h. The mitochondrial stress test was then performed as described in Materials and Methods, *n*=4–6, each experiment was performed three times. Error bars represent S.E.M., *P<0.05 max OCR *versus* basal OCR for AICAR-treated cells in glucose+palmitate (G+P), at the time point indicated; ^#^*P*<0.05 max OCR for AICAR treated *versus* max OCR for untreated in G+P, at the time point indicated. (**d**) Neonatal rat cardiac myocytes were cultured in complete growth medium treated with vehicle or 500 *μ*M AICAR in the presence of adenoviral-control shRNA or -shRNA-Ppar*α*- constructs, for 24 h. The medium was then replaced with serum-free XF medium containing 17.5 mM glucose plus 100 *μ*M palmitate-BSA containing vehicle or 500 *μ*M AICAR, for 1 h. The mitochondrial stress test was then performed as described in Materials and Methods, *n*=4–6, each experiment was performed two times. Error bars represent S.E.M., *P<0.05 max OCR *versus* basal OCR for AICAR-treated cells, at the time point indicated; ^#^*P*<0.05 max OCR for AICAR treated *versus* max OCR for control; ^$^*P*<0.05 max OCR for AICAR treated *versus* max OCR of AICAR- plus shRNA-Ppar*α*-treated, at the time point indicated. (**e** and **f**) Neonatal cardiac myocytes were treated as described in (**a** and **b**), with AICAR in normoxia *versus* hypoxia/reoxygenation (hypox/reox), as indicated. Total mRNA (**e**) and DNA (**f**) were extracted and subjected to qPCR for the indicated genes (*n*=4). The results were averaged and plotted as relative values to control medium (no glucose or fatty acids). Error bars represent S.E.M., *P<0.05 *versus* control, ^#^*P*<0.05 *versus* control hypox/reoxx. (**g**) Neonatal myocyes were treated with adenoviral shRNA-control and shRNA-Ppar*α*, with or without 500 *μ*M AICAR for 24 h in the presence of 17.5 mM glucose+100 *μ*M palmitate-BSA. Total RNA was extracted and analyzed by qPCR for the indicated genes. The results were averaged and plotted as values relative to control. Error bars represent S.E.M., *P<0.05 *versus* control

AMPK is a metabolic sensor^19^ that can acutely enhance fatty acid oxidation via an increase in acetyl-CoA carboxylase 2 phosphorylation-mediated activation of Cpt1.^20^ Thus, we hypothesized that AMPK, through activating Cpt1 may contribute to RRC and/or basal OCR during recovery from hypoxia. To test this, we treated the myocytes with either an adenosine analog activator of AMPK (5-amino-1-*β*-D-ribofuranosyl-imidazole-4-carboxamide (AICAR)) or AICAR plus the AMPK inhibitor, compound C (CC). The results show that AICAR increased reserve from 1.34-fold to 1.9-fold, but had no effect on basal OCR during normoxia (Figure 3a, upper panel). The addition of CC inhibitor completely abolished the effect of AICAR on reserve, and like etomoxir, had no effect on basal OCR, thus, confirming that the effects of AICAR are indeed AMPK dependent. A similar effect was seen after hypoxia/reoxygenation, where the RRC was 2.5-fold *versus* basal OCR (Figure 3b, upper panel). However, unlike what we have seen with DCA in Figure 2, AICAR did not rescue the reduction in basal OCR. Moreover, AICAR's effect on RRC was only elicited in the presence of glucose plus palmitate, but not palmitate alone (Figure 3c, upper panel). This is expected in the neonatal myocytes, as palmitate strictly requires the presence of glucose to enhance RRC.

Since the time point used in the latter experiments for activation AMPK is 48 h, there is a possibility that the effects of AICAR are mediated through a transcriptional, peroxisome proliferator-activated receptor alpha (Ppar*α*)-dependent, mechanism. qPCR results confirm that AICAR enhanced the expression of Ppar*α*, peroxisome proliferator-activated receptor gamma, coactivator 1 alpha (Pgc-1*α*), and Cpt1 during normoxia (Figure 3e). The same set of genes was also induced by hypoxia, however, 24 h post hypoxia, while Pgc-1*α* and Cpt1 remained higher than control, Ppar*α* was significantly lower, and recovered by treatment with AICAR (Figure 3e). A pattern similar to Ppar*α* expression was seen with transcription factor A, mitochondrial (Tfam) and mitochondrial numbers (Figure 3f). Therefore, to determine the role of Ppar*α* in mediating the effect of AICAR on RRC, we measured OCR in the presence of short hairpin RNA (sh) targeting Ppar*α* (Ppar*α*-sh). Treatment with this construct resulted in complete inhibition of AICAR-induced upregulation of Ppar*α* (~0.5-fold of basal), and a 3.5-fold increase in Pgc-1*α* mRNA (mechanism unknown), but had no effect on Cpt1 or Tfam (Figure 3g). This was accompanied by 70% inhibition of AICAR-induced RRC (Figure 3d, upper panel). The results show that after hypoxia, fatty acid oxidation may be compromised due to a reduction in Ppar*α*, and that this could be prevented by AMPK activation, resulting in restoration of RRC.

### Regulation of the reserve respiratory capacity in human iPSC-derived cardiac myocytes

**Figure 4:**
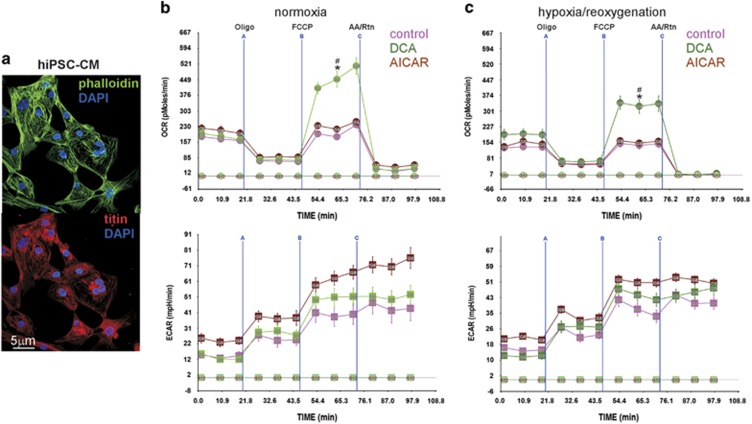
The RRC in human iPSC-CM is regulated by DCA but not by AICAR. (**a**) Human iPSC-CM were cultured, fixed, permeabilized and incubated with the indicated primary antibodies. They were then incubated with fluorescently labeled phalliodin (upper panel) or secondary antibody (lower panel), mounted with DAPI and imaged by confocal microscopy. (**b** and **c**) Human iPSC-CM were cultured in complete growth medium containing vehicle, 1 mM DCA or 500 *μ*M AICAR and either remained in normoxic conditions (atmospheric O_2_) (**b**) or were exposed to hypoxia (<1% O_2_) (**c**), for 24 h. At the end of this period, the medium was changed to base medium containing 17.5 mM glucose plus 100 *μ*M palmitate-BSA containing vehicle, 1 mM DCA or 500 *μ*M AICAR, as indicated, for an additional 24 h. The medium was then replaced with serum-free XF medium containing 17.5 mM glucose plus 100 *μ*M palmitate-BSA containing vehicle, 1 mM DCA or 500 *μ*M AICAR, for 1 h. The mitochondrial stress test was then performed as described in Materials and Methods, *n*=4–6, each experiment was performed twice. Error bars represent S.E.M., *P<0.05 max OCR *versus* basal OCR for DCA-treated cells, at the time point indicated; ^#^*P*<0.05 max OCR for DCA-treated *versus* max OCR for control, at the time point indicated

The hiPSC-CM are >95% differentiated myocytes that are spontaneously beating in culture (Figure 4a). As shown in Figure 1, these cells have an RRC that is dependent on fatty acid only and is dampened by glucose. So while the neonatal myocytes benefit from the addition of DCA as it enhances both basal OCR and RRC, it was unclear how the hiPSC-CM would benefit from an increase in glucose oxidation. Interestingly, our results show that DCA increases RRC before (~2.5-fold, Figure 4b, upper panel) and after hypoxia (~1.7-fold, Figure 4c, upper panel). The treatment had little impact on basal OCR during normoxia, but restored the decrease seen after hypoxia. In contrast, AICAR did not have a significant effect on the OCR in these cells, plausibly due to inherent optimal fatty acid oxidation. On the other hand, AICAR enhanced ECAR, indicating an increase in glycolysis (Figures 4b and c, lower panels). These results indicate that activation of pyruvate dehydrogenase in either neonatal cardiac myocytes or hiPSC-CM has the capacity to significantly increase RRC and, thereby, restore the cells' bioenergetics after exposure to hypoxia.

### Complex II is a source of RRC

**Figure 5:**
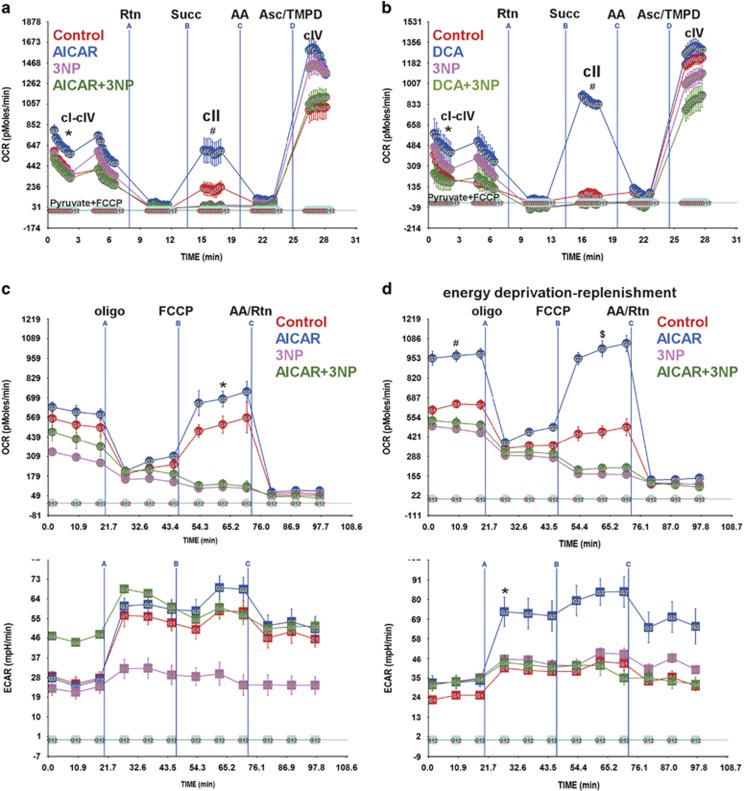

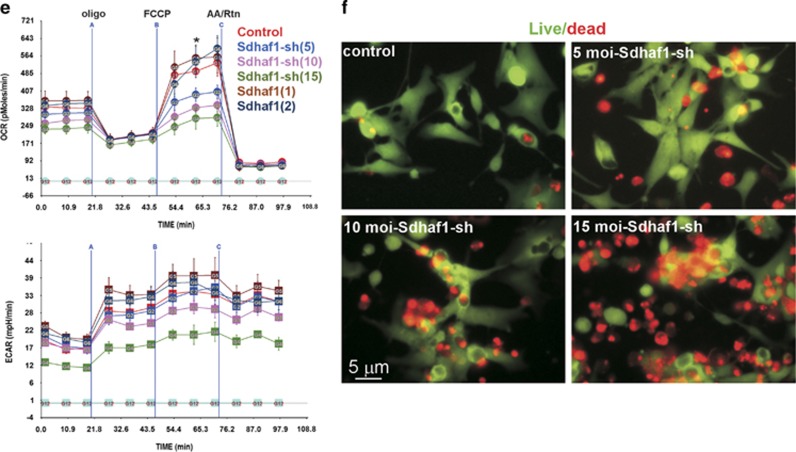
RRC is a product of cII activity and is utilized after energy deprivation. (**a** and **b**) Neonatal rat cardiac myocytes cultured in complete growth medium were treated with vehicle, 500 *μ*M AICAR (**a**) or 1 mM DCA (**b**), for 24 h. Vehicle or 100 *μ*M 3NP was added for the last 1 h of this incubation. The cells were then permeabilized with 0.5 nM PMP reagent (rPFO) in the presence of 10 mM pyruvate, 2 mM malate and 4 *μ*M FCCP, for 30 min. The electron flow assay was then performed as described in Materials and Methods, as indicated, *n*=4–6, error bars represent S.E.M., each experiment was performed twice. Error bars represent S.E.M., *P<0.05 basal OCR for AICAR or DCA treated *versus* basal OCR for control, at the time point indicated; ^#^*P*<0.05 max OCR for AICAR or DCA treated *versus* max OCR for control, at the time point indicated. (**c** and **d**) Neonatal rat cardiac myocytes cultured in complete growth medium were treated with vehicle or 500 *μ*M AICAR, for 24 h. Vehicle or 100 *μ*M 3NP was added and the cells either remained in normoxic conditions (atmospheric O_2_) (**c**) or were exposed to hypoxia (<1% O_2_) and glucose deprivation (**d**), for 1 h. At the end of this period, the medium was then replaced with serum-free XF medium containing 17.5 mM glucose plus 100 *μ*M palmitate-BSA containing vehicle, 500 *μ*M AICAR, 100 *μ*M 3NP or 500 *μ*M AICAR plus 100 *μ*M 3NP, for 1 h. The mitochondrial stress test was then performed as described in Materials and Methods, *n*=4–6, each experiment was performed twice. Error bars represent S.E.M., *P<0.05 max OCR *versus* basal OCR for AICAR-treated cells, at the time point indicated; ^#^*P*<0.05 basal OCR for AICAR treated *versus* basal OCR for control, at the time point indicated; ^$^*P*<0.05 max OCR for AICAR treated *versus* max OCR for control, at the time point indicated. (**e**) Neonatal rat cardiac myocytes (50 000–100 000/well) cultured in complete growth medium were infected with adenoviral vectors harboring a scrambled control sequence, shRNA targeting Sdhaf1 (5, 10, 15 moi) (Sdhaf1-sh), or an Sdhaf1 overexpressor (1, 2 moi), for 24 h. The medium was then replaced with serum-free XF medium containing 17.5 mM glucose plus 100 *μ*M palmitate-BSA, for 1 h. The mitochondrial stress test was then performed as described in Materials and Methods, *n*=3–4. Error bars represent S.E.M., *P<0.05 max OCR control *versus* max OCR for Sdhaf1-sh treated (all doses), at the time point indicated. (**f**) Cells treated as described in (**e**) were subjected to a live (green)/dead (red nuclei) assay and imaged

In an attempt to identify the source of RRC in the cell, we measured the activities of the respiratory complexes in an electron flow assay. The results reveal that AICAR induced an overall increase in electron flow activity in the uncoupled state (1.5- to 1.7-fold), and, in specific, an increase in cII activity (1.6- to 2-fold). The cII-specific inhibitor 3-nitropropionate (3NP) completely abolished the effect of AICAR on basal flow (cI-cIV), restoring it to baseline values, while completely suppressing cII activity (Figure 5a). A similar effect was observed with DCA (Figure 5b). Notably, complex IV activity varied according to changes in electron flow. It should also be noted that there were quantitative but not qualitative differences in basal and activated cII OCR values between the primary myocyte cultures, as observed in Figures 5a and b. Consequently, we predicted that the RRC might be a product of an increase in complex II activity. To test this, we measured the effect of AICAR on RRC in the presence or absence of 3NP. As demonstrated in Figure 5c, 3NP completely abolished RRC, in addition to, minimal (15–20%), but insignificant, effect on basal OCR. Thus, we concluded that an increase in cII activity contributes to formation of RRC via increasing electron flow directly from FADH_2_-succinate dehydrogenase.

We speculated that after a short period of nutrient and oxygen deprivation and before cell demise (as assessed by a live/dead cell assay, Supplementary Figure S2), myocytes will attempt to recover their energy supply by increasing energy production. In accordance, AMPK, through activation of cII, will increase basal OCR and oxidative phosphorylation. This was confirmed after incubating the cells for 1 h in <1% O_2_ with no glucose, where pre-incubation with AICAR induced almost a 2-fold increase in basal OCR and oxidative phosphorylation (the difference between OCR before and after addition of oligomycin) *versus* control, which were completely abolished by cII inhibition, as they were reduced to control values (Figure 5d, upper panel). In addition, AICAR significantly enhanced ECAR (Figure 5d, lower panel). Thus, cII harbors reserve activity that can be activated by AMPK, which allows the mitochondria to rapidly increase its energy output after energy deprivation.

To validate the results of the 3NP inhibitor on cII and RRC, we knocked down one of cII's assembly factors, succinate dehydrogenase assembly factor 1 (Sdhaf1). Figure 5e shows that knockdown of Sdhaf1 with a short hairpin (sh) RNA construct (Sdhaf1-sh) completely abrogated the RRC, with no significant effect on basal OCR at the lowest dose used of the adenoviral delivery vector (multiplicity of infection, moi 5) (Figure 5e). Increasing doses of Sdhaf1-sh, however, resulted in significant reduction (20–35%) of basal OCR that was associated with almost an equivalent degree of cell death (Figure 5f). Since unassembled Sdha is a known source of excessive ROS formation, it explains the cell death observed at the higher doses of Sdhaf1-sh. The results confirm that holo-cII is indeed required for formation of RRC.

### RRC and cII contribute to an increase in cell survival after energy deprivation

**Figure 6:**
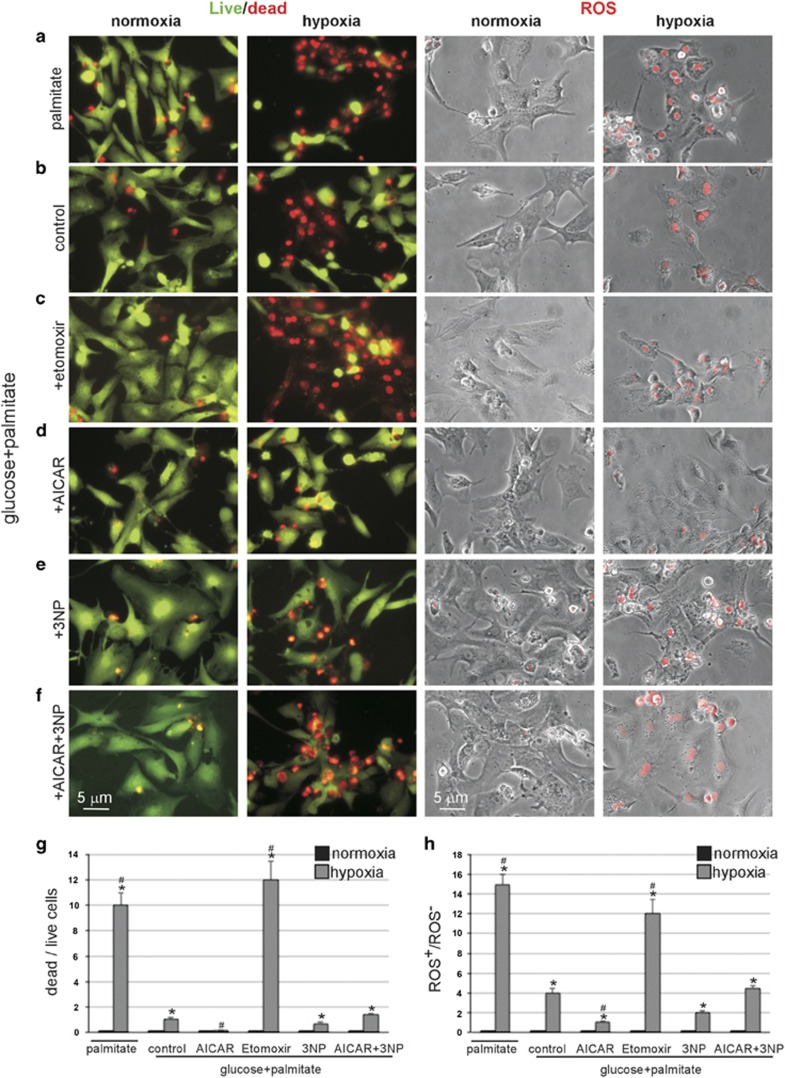
Conditions that are associated with a higher RRC are more resistant to cell death and produce less ROS. Cardiac myocytes were incubated in glucose+palmitate (**b**–**f**) except in (**a**) in palmitate only, (**c**)+etomoxir (Cpt1 inhibitor), (**d**) +AICAR (AMPK activator), for 24 h in normoxic conditions, (**e**) +3NP (cII inhibitor), for 1 h before hypoxia, (**f**) +AICAR+3NP (left and third from left panels **a**–**f**). Cells were then subjected to 6 h of <0% O_2_ for 6 h in the absence of glucose (second from the left and right panels **a**–**f**). This was followed by a live (green)/dead (red nuclei) assay (left and second from the left panels **a**–**f**) or ROS detection (red nuclei, right and third from the left panels **a**–**f**) that was visualized by live imaging of the cells. (**g**) The dead/live cell ratio was calculated and plotted (*n*=3, 100 cells counted). Error bars represent S.E.M. and *P<0.05 *versus* normoxia. ^#^*P*<0.05 *versus* hypoxia (glucose+palmitate). (**h**) The ROS^+^/ROS^−^ cell ratio was calculated and plotted (*n*=2, 60 cells counted). Error bars represent S.E.M. and *P<0.05 *versus* normoxia. ^#^*P*<0.05 *versus* hypoxia (glucose+palmitate)

The RRC has been shown to correlate with cell survival in multiple cell types.^1, 2^ Our goal here was to determine whether the different regulators of RRC that we have identified in myocytes had an effect on cell survival and whether cII had any role in the process. To address this, we incubated the cells with palmitate-BSA only, or glucose plus palmitate in the absence or presence of etomoxir, AICAR, 3NP or 3NP plus AICAR, before subjecting them to <1% O_2_ conditions in the absence of glucose for 6 h (lethal conditions). Significantly, in the presence of atmospheric O_2_ none of these treatments had an effect on cell survival or ROS production (Figures 6a–f, first and third panels from the left). After energy deprivation, followed by restoration for 30 min, cell death was >90%, accompanied by ROS production, when either glucose or fatty acid oxidation was inhibited glucose deprivation or etomoxir treatment, respectively (Figures 6a and c, second and fourth panels from the left, and Figure 6g), *versus* ~10% in control cells with glucose+palmitate (Figures 6b and g). Conversely, cells treated with AICAR exhibited <1% cell death (Figure 6d, second panel and fourth panels from the left, and Figure 6g), which was lost by treatment with the cII inhibitor 3NP (Figure 6f, second and fourth panels from the left, and Figure 6g). Here, we used the membrane-impermeable propidium iodide staining for detection of nuclei in dead cells,^21, 22^ which is dependent on a damaged plasma membrane for cell entry. This suggests that the mode of cell death detected in this experiment is mainly necrotic. We, thus, conclude that activation of cII and formation of an RRC allows the cells to minimize ROS formation and resist cell death due to energy deprivation, plausibly by increasing energy production after restoration of oxygen and nutrients, as seen in Figure 5.

### Sirt3 is required for the development of RRC

**Figure 7:**
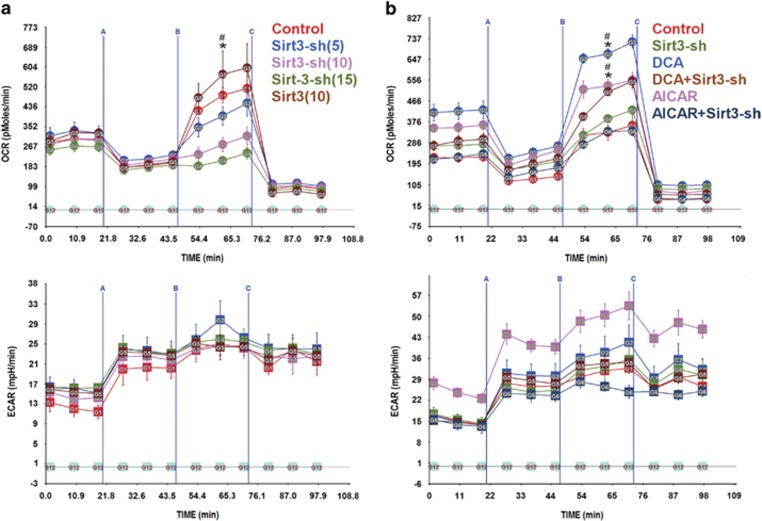
Sirt3 is required for development of RRC. (**a**) Neonatal rat cardiac myocytes cultured in complete growth medium were infected with adenoviral vectors harboring a scrambled control sequence, shRNA targeting Sirt3 (Sirt3-sh) (5, 10 or 15 moi) or a Sirt3 overexpressor (10 moi), for 24 h. The medium was then replaced with serum-free XF medium containing 17.5 mM glucose plus 100 *μ*M palmitate-BSA, for 1 h. The mitochondrial stress test was then performed as described in Materials and Methods, *n*=3–4. Error bars represent S.E.M., *P<0.05 max OCR *versus* basal OCR for control, at the time point indicated; ^#^*P*<0.05 max OCR for control *versus* max OCR for Sirt3-sh treated (10 and 15 moi), at the time point indicated. (**b** and **c**) Neonatal rat cardiac myocytes cultured in complete growth medium were treated with 500 *μ*M AICAR (**b**) or 1 mM DCA (**c**) in the presence of an adenoviral vector harboring a scrambled control sequence or a shRNA targeting Sirt3 (Sirt3-sh) (10 moi), for 24 h. The medium was then replaced with serum-free XF medium containing 17.5 mM glucose plus 100 *μ*M palmitate-BSA, 17.5 mM glucose plus 100 *μ*M palmitate-BSA plus 1 mM DCA or 17.5 mM glucose plus 100 *μ*M palmitate-BSA plus 500 *μ*M AICAR, for 1 h. The mitochondrial stress test was then performed as described in Materials and Methods, *n*=3–4. Error bars represent S.E.M., *P<0.05 max OCR *versus* basal OCR for DCA- or AICAR-treated cells, at the time point indicated; ^#^*P*<0.05 max OCR for DCA or AICAR treated *versus* max OCR for DCA+Sirt3-sh or AICAR+Sirt3-sh treated, at the time point indicated

Sirt3 is a mitochondrial sirtuin that regulates a plethora of proteins including NDUFA9 and complex I,^23^ as well as, complex II activity.^24, 25^ We predicted that the effects of AMPK and PDH that regulate substrate flux into the TCA cycle and development of RRC might be mediated through the NAD^+^-dependent Sirt3. To test this, we generated a short hairpin RNA targeting Sirt3 (Sirt3-sh). This construct, dose-dependently, abolished the RRC in cardiac myocytes, but had no effect on basal OCR (Figure 7a). Conversely, overexpressing Sirt3 resulted in a slight, but not significant increase in RRC, possibly due to saturating endogenous levels under these conditions. The results demonstrate that Sirt3 is required for the development of RRC, but has minimal effect on basal OCR. In agreement, Sirt3 knockdown also inhibited AICAR- and DCA-induced RRC (Figure 7b).

## Discussion

A cell's energy production is directly linked to its demand.^26, 27^ In this report, we show that cardiac myocytes operate on a fraction of their mitochondrial respiratory capacity under conditions where the preferred metabolic substrates are available. Although neonatal cardiac myocytes utilize glucose as the preferred substrate, a respiratory reserve will only develop in the presence of both glucose and fatty acids in this cell type, which suggests independent resources for basal OCR *versus* the RRC. We also show here, for the first time, that we can further increase the RRC by enhancing either glucose or fatty acid oxidation, via inhibiting pyruvate dehydrogenase kinases or stimulation of AMPK-Ppar*α* axis, respectively. Conversely, hypoxia exhausts the RRC, which can be rescued by those measures. This suggested that the RRC is a product of a co-regulated increase in the TCA cycle and electron transport chain (ETC) activities.

Succinate dehydrogenase is the only enzyme in the TCA cycle that is also a component of the electron transport chain complex II, which suggested to us that it is well positioned to serve as a regulated source of the RRC. Indeed, our data show that an increase in glucose and/or fatty acid oxidation is sensed by cII, resulting in an increase in its activity. However, this spare cII activity that was recently observed^28^ remains unutilized under normal conditions, but becomes manifest when oxygen consumption is uncoupled from ATP production (Figures 1, 2, 3, 4), or if there is an increase in energy demand (Figure 5). We show that inhibition of the Sdha subunit of cII by 3NP completely abrogates the RRC, proving that it is indeed a product of cII activity. These results were corroborated by a second independent approach that involved the knockdown of the assembly factor Sdhaf1, which, similarly, completely abolished RRC before it induced cell death. The cell death observed with Sdhaf1 knockdown is an expected effect of the excessive ROS produced by the disassembled Sdha subunit.^16^ Interestingly, in both approaches, the inhibition of cII resulted in only minimal inhibition of basal OCR (10–18%), which is equivalent to the extent that cII contributes to the transfer of electrons from FADH and its oxidation via the ETC. Finally, we show that Sirt3 is required for formation of RRC, suggesting that it also regulates cII activity. This is indeed supported by the fact that Sdha has 13 lysine-acetylation sites that are deacetylated by Sirt3, and which regulate its activity.^24, 25^

Sdhaf1 gene has been recently discovered in association with infantile leukoencephalopathy, an infantile mitochondrial disease, where it harbors a missense point mutation,^29^ underscoring its critical regulatory function. This mutation is associated with a reduction in the cII holoenzyme (assembled complex) and an ~70–80% loss in Sdh and succinyl CoQ reductase activities in fibroblasts and muscle cells. Our data confirm that Sdhaf1 is critical for cII function in cardiac myocytes, as it is required for development of RRC, which enhances cell survival.

In summary, the work presents a novel mechanism by which cells can increase their energy output during an increase in demand, which includes assembly and activation of cII by the metabolic sensors and regulators, AMPK, PDH and Sirt3. In addition, we show that this parameter can be exploited to enhance the cell's tolerance to cell death signals.

## Materials and Methods

### Neonatal rat cardiac myocyte culture

Cardiac myocytes were prepared from 1- to 2-day-old Sprague–Dawley rats as previously described.^30^ The myocytes were treated in Dulbecco's modified essential medium/Ham F12 (1 : 1) supplemented with 10% fetal bovine serum (10% FBS) and 1% penicillin/streptomycin or in Dulbecco's modified essential medium (1 ×) supplemented with glucose or palmitate-BSA, as indicated in the figure legends. Pharmacological treatments include etomoxir, dichloroacetate (DCA), Aicar and 3 nitropropionate (3NP) purchased from Sigma-Aldrich Corp. (St. Louis, MO, USA).

### Human iPSC-cardiac myocyte culture

Human iCell cardiac myocytes (Cellular Dynamics International, Madison, WI, USA) were thawed and plated, as recommended by the manufacturer. After 48 h, the medium was changed to Dulbecco's modified essential medium/Ham F12 (1 : 1) supplemented with 10% FBS and 1% penicillin/streptomycin. The cells were treated in this medium or in Dulbecco's modified essential medium (1 ×) supplemented with glucose or palmitate-BSA as indicated in the figure legends.

### Adenovirus constructs

The human cDNA clones for Pdk1 (SC321678/NM_002610), Pdk4 (SC118542/NM_002612), Sdhaf1 (NM_001042631) and Sirt3 (NM_012239) were purchased (Origene Technologies, Inc., Rockville, MD, USA) and cloned into recombinant adenovirus vectors, propagated, and titered as previously described by Graham and Prevec.^31^

Short hairpin constructs targeting succinate dehydrogenase assembly factor 1 (*Sdhaf1*) nt 31–50 of *Rattus norvegicus* NM_001177687.1 and *Sirt3* nt 293–312 of *Rattus norvegicus* NM_001106313.2 were designed, synthesized (IDT), and cloned into adenoviral vectors. Cardiac myocytes were treated as indicated in the figure legends at an moi of 1–15.

### Hypoxia

Neonatal rat cardiac myocytes or human iPSC-CM were subjected to hypoxia using a hypoxic chamber (Billups-Rothenberg, Inc., Del Mar, CA, USA) or (BioSpherix, Lacona, NY, USA). Either chamber is perfused with a gas mixture of 95% N_2_ and 5% CO_2_ at 7 psi/12 000 kPa filling pressure, until a chamber oxygen concentration of 0.1% is reached. The chamber was then kept in a 37 °C incubator.

### Mitochondrial stress test

Neonatal cardiac myocytes (50 000–100 000 cells/well) or human iPS-derived cardiac myocytes (60 000/well) were plated in 24-well Seahorse analyzer plates. OCR and ECAR were measured using the Seahorse XF24-3 Analyzer (Seahorse Bioscience, North Billerica, MA, USA), as recommended by the manufacturer. Briefly, after 20 (A), 45 (B), 70 (C) min of the start of measurements, the medium was injected with A, oligomycin; B, FCCP; C, antimycin A plus rotenone, as marked in the graphs. Three readings were taken after each injected compound and the results plotted as pMoles/min/well or mpH/min/well (y axis) *versus* time (x axis).

### Electron flow assay

OCR was measured in cardiac myocytes after cell permeabilization using XF PMP reagent (Seahorse Bioscience), as recommended by the manufacturer. Briefly, 10 mM pyruvate plus 2 mM malate are used as substrates, in addition to 4 *μ*M FCCP. Final concentrations of injected compounds were as follows: port A, 2 *μ*M rotenone; port B, 10 mM succinate; port C, 4 *μ*M antimycin A; port D, 10 mM ascorbate plus 0.1 mM TMPD.

### Immunocytochemistry

Neonatal rat cardiac myocytes or human iPSC-CM were plated on gelatin-coated glass slides and treated, as indicated in the figure legends. The cells were then fixed, permeabilized, immunolabelled, mounted, and imaged by confocal microscopy. Anti-OxPhos Complex IV (Life Technologies, Grand Island, NY, USA) and anti-titin (Hybridoma Bank, University of Iowa, Iowa City, IA, USA) antibodies were used.

### Quantitative PCR

Total RNA or DNA was extracted using TRIzol Reagent (Life Technologies). RNA was reversed transcribed to cDNA using High Capacity cDNA Reverse Transcription Kit (Applied Biosystems, a brand of Life Technologies). Quantitative PCR was performed using TaqMan gene expression assays (Applied Biosystems) on Applied Biosystems 7500 Real-Time PCR system for the following genes: 18S (Mm03928990_g1), Pdk1 (Rn00587598_m1), Pdk2 (Rn00446679_m1), Pdk3 (Rn01424337_m1), Pdk4 (Rn00585577_m1), Ppargc1 (Mm01208835_m1), PPARa (Rn00566193_m1), Cpt1 (Rn00682395_m1), tfam (Rn00580051_m1) and ND2 (Rn03296765_S1). Mitochondrial DNA was quantified using a primer/probe set for the mitochondrial D-loop (Applied Biosystems), as described by Nicklas *et al.*^32^

### Live/dead cell assay

Neonatal rat cardiac myocytes were plated on glass slides and treated, as indicated in the figure legends. Live/dead cells assay were performed using the LIVE/DEAD Viability/Cytotoxicity Kit (Life Technologies).

### Superoxide assay

Neonatal rat cardiac myocytes were plated on glass slides and treated, as indicated in the figure legends. Superoxide production was measured using MitoSOX Red mitochondrial superoxide indicator (Life Technologies).

### Statistical analysis

Significant differences between the mean of two sample groups were calculated using *T*-test (equal variance, two-tailed), where *P*<0.05 was considered as significant.

## Abbreviations

3NP: 3-nitropropionate
ACC: acetyl CoA carboxylase
AICAR: 5-aminoimidazole-4-carboxamide-1-*β*-D-ribofuranosyl 5'-monophosphate
AMPK: AMP-activated protein kinase
ATP: adenosine triphosphate
BSA: bovine serum albumin
cII: complex II
CC: compound C
CPT1: carnitine palmitoyltransferase 1
DAPI: 4',6-diamidino-2-phenylindole
DCA: dichloroacetate
DMEM: Dulbecco's Modified Essential Medium
ECAR: extracellular acidification rate
ETC: electron transport chain
FAD: flavin adenine dinucleotide
FBS: fetal bovine serum
FCCP: carbonyl cyanide 4-(trifluoromethoxy)phenylhydrazone
GAPDH: glyceraldehyde-3-phosphate dehydrogenase
Hif-1*α*: hypoxia inducible factor 1 alpha
iPSC-CM: induced pluripotent stem cell-derived cardiac myocyte
moi: multiplicity of infection
NAD: nicotinamide adenine dinucleotide
OCR: oxygen consumption rate
OXPHOS: oxidative phosphorylation
PBS: phosphate-buffered saline
PDH: pyruvate dehydrogenase
PDK: pyruvate dehydrogenase kinase
PPAR: peroxisome proliferator activated receptor
ROS: reactive oxygen species
RRC: reserve respiratory capacity
SDH: succinate dehydrogenase
SDHAF: succinate dehydrogenase assembly factor
SEM: standard error of the mean
shRNA: short hairpin ribonucleic acid
SIRT: sirtuin

## References

[bib1] 1Nickens KP, Wikstrom JD, Shirihai OS, Patierno SR, Ceryak S. A bioenergetic profile of non-transformed fibroblasts uncovers a link between death-resistance and enhanced spare respiratory capacity. Mitochondrion 2013; 13: 662–667. 24075934 10.1016/j.mito.2013.09.005 PMC3837383

[bib2] 2Yadava N, Nicholls DG. Spare respiratory capacity rather than oxidative stress regulates glutamate excitotoxicity after partial respiratory inhibition of mitochondrial complex I with rotenone. J Neurosci 2007; 27: 7310–7317. 17611283 10.1523/JNEUROSCI.0212-07.2007 PMC6794596

[bib3] 3Choi SW, Gerencser AA, Nicholls DG. Bioenergetic analysis of isolated cerebrocortical nerve terminals on a microgram scale: spare respiratory capacity and stochastic mitochondrial failure. J Neurochem 2009; 109: 1179–1191. 19519782 10.1111/j.1471-4159.2009.06055.x PMC2696043

[bib4] 4Flynn JM, Choi SW, Day NU, Gerencser AA, Hubbard A, Melov S. Impaired spare respiratory capacity in cortical synaptosomes from Sod2 null mice. Free Radic Biol Med 2011; 50: 866–873. 21215798 10.1016/j.freeradbiomed.2010.12.030 PMC3061438

[bib5] 5Hill BG, Benavides GA, Lancaster JRJr, Ballinger S, Dell'Italia L, Jianhua Z et al. Integration of cellular bioenergetics with mitochondrial quality control and autophagy. Biol Chem 2012; 393: 1485–1512. 23092819 10.1515/hsz-2012-0198 PMC3594552

[bib6] 6Nicholls DG. Spare respiratory capacity, oxidative stress and excitotoxicity. Biochem Soc Trans 2009; 37: 1385–1388. 19909281 10.1042/BST0371385

[bib7] 7Sansbury BE, Jones SP, Riggs DW, Darley-Usmar VM, Hill BG. Bioenergetic function in cardiovascular cells: the importance of the reserve capacity and its biological regulation. Chem Biol Interact 2011; 191: 288–295. 21147079 10.1016/j.cbi.2010.12.002 PMC3090710

[bib8] 8Siddiqui A, Rivera-Sanchez S, Castro Mdel R, Acevedo-Torres K, Rane A, Torres-Ramos CA et al. Mitochondrial DNA damage is associated with reduced mitochondrial bioenergetics in Huntington's disease. Free Radic Biol Med 2012; 53: 1478–1488. 22709585 10.1016/j.freeradbiomed.2012.06.008 PMC3846402

[bib9] 9van der Windt GJ, Everts B, Chang CH, Curtis JD, Freitas TC, Amiel E et al. Mitochondrial respiratory capacity is a critical regulator of CD8+ T cell memory development. Immunity 2012; 36: 68–78. 22206904 10.1016/j.immuni.2011.12.007 PMC3269311

[bib10] 10Bourgeron T, Rustin P, Chretien D, Birch-Machin M, Bourgeois M, Viegas-Pequignot E et al. Mutation of a nuclear succinate dehydrogenase gene results in mitochondrial respiratory chain deficiency. Nat Genet 1995; 11: 144–149. 7550341 10.1038/ng1095-144

[bib11] 11Jain-Ghai S, Cameron JM, Al Maawali A, Blaser S, MacKay N, Robinson B et al. Complex II deficiency—a case report and review of the literature. Am J Med Genet A 2013; 161A: 285–294. 23322652 10.1002/ajmg.a.35714

[bib12] 12Walker DW, Hajek P, Muffat J, Knoepfle D, Cornelison S, Attardi G et al. Hypersensitivity to oxygen and shortened lifespan in a Drosophila mitochondrial complex II mutant. Proc Natl Acad Sci USA 2006; 103: 16382–16387. 17056719 10.1073/pnas.0607918103 PMC1618815

[bib13] 13Wojtovich AP, Brookes PS. The complex II inhibitor atpenin A5 protects against cardiac ischemia-reperfusion injury via activation of mitochondrial KATP channels. Basic Res Cardiol 2009; 104: 121–129. 19242645 10.1007/s00395-009-0001-y PMC2776710

[bib14] 14Albayrak T, Grimm S. A high-throughput screen for single gene activities: isolation of apoptosis inducers. Biochem Biophys Res Commun 2003; 304: 772–776. 12727223 10.1016/s0006-291x(03)00653-3

[bib15] 15Grimm S. Respiratory chain complex II as general sensor for apoptosis. Biochim Biophys Acta 2013; 1827: 565–572. 23000077 10.1016/j.bbabio.2012.09.009

[bib16] 16Lemarie A, Huc L, Pazarentzos E, Mahul-Mellier AL, Grimm S. Specific disintegration of complex II succinate:ubiquinone oxidoreductase links pH changes to oxidative stress for apoptosis induction. Cell Death Differ 2011; 18: 338–349. 20706275 10.1038/cdd.2010.93 PMC3044456

[bib17] 17Quinlan CL, Orr AL, Perevoshchikova IV, Treberg JR, Ackrell BA, Brand MD. Mitochondrial complex II can generate reactive oxygen species at high rates in both the forward and reverse reactions. J Biol Chem 2012; 287: 27255–27264. 22689576 10.1074/jbc.M112.374629 PMC3411067

[bib18] 18Whitehouse S, Cooper RH, Randle PJ. Mechanism of activation of pyruvate dehydrogenase by dichloroacetate and other halogenated carboxylic acids. Biochem J 1974; 141: 761–774. 4478069 10.1042/bj1410761 PMC1168183

[bib19] 19Zaha VG, Young LH. AMP-activated protein kinase regulation and biological actions in the heart. Circ Res 2012; 111: 800–814. 22935535 10.1161/CIRCRESAHA.111.255505 PMC4397099

[bib20] 20Dobrzyn P, Dobrzyn A, Miyazaki M, Cohen P, Asilmaz E, Hardie DG et al. Stearoyl-CoA desaturase 1 deficiency increases fatty acid oxidation by activating AMP-activated protein kinase in liver. Proc Natl Acad Sci USA 2004; 101: 6409–6414. 15096593 10.1073/pnas.0401627101 PMC404058

[bib21] 21Regula KM, Baetz D, Kirshenbaum LA. Nuclear factor-kappaB represses hypoxia-induced mitochondrial defects and cell death of ventricular myocytes. Circulation 2004; 110: 3795–3802. 15596562 10.1161/01.CIR.0000150537.59754.55

[bib22] 22Regula KM, Ens K, Kirshenbaum LA. Inducible expression of BNIP3 provokes mitochondrial defects and hypoxia-mediated cell death of ventricular myocytes. Circ Res 2002; 91: 226–231. 12169648 10.1161/01.res.0000029232.42227.16

[bib23] 23Abdellatif M. Sirtuins and pyridine nucleotides. Circ Res 2012; 111: 642–656. 22904043 10.1161/CIRCRESAHA.111.246546 PMC3496429

[bib24] 24Cimen H, Han MJ, Yang Y, Tong Q, Koc H, Koc EC. Regulation of succinate dehydrogenase activity by SIRT3 in mammalian mitochondria. Biochemistry 2010; 49: 304–311. 20000467 10.1021/bi901627u PMC2826167

[bib25] 25Finley LW, Haas W, Desquiret-Dumas V, Wallace DC, Procaccio V, Gygi SP et al. Succinate dehydrogenase is a direct target of sirtuin 3 deacetylase activity. PLoS One 2011; 6: e23295. 21858060 10.1371/journal.pone.0023295 PMC3157345

[bib26] 26van Beek JH. Adenine nucleotide-creatine-phosphate module in myocardial metabolic system explains fast phase of dynamic regulation of oxidative phosphorylation. Am J Physiol Cell Physiol 2007; 293: C815–C829. 17581855 10.1152/ajpcell.00355.2006

[bib27] 27Vendelin M, Lemba M, Saks VA. Analysis of functional coupling: mitochondrial creatine kinase and adenine nucleotide translocase. Biophys J 2004; 87: 696–713. 15240503 10.1529/biophysj.103.036210 PMC1304393

[bib28] 28Wust RC, Helmes M, Stienen GJ. Rapid changes in NADH and flavin autofluorescence in rat cardiac trabeculae reveal large mitochondrial complex II reserve capacity. J Physiol 2015; 593: 1829–1840. 25640645 10.1113/jphysiol.2014.286153 PMC4405745

[bib29] 29Ghezzi D, Goffrini P, Uziel G, Horvath R, Klopstock T, Lochmuller H et al. SDHAF1, encoding a LYR complex-II specific assembly factor, is mutated in SDH-defective infantile leukoencephalopathy. Nat Genet 2009; 41: 654–656. 19465911 10.1038/ng.378

[bib30] 30Abdellatif M, Mcllelan WR, Schneider MD. p21 Ras as a governer of global gene expression. J Biol Chem 1994; 269: 15423–15426. 8195182

[bib31] 31Graham FL, Prevec L. Methods in Molecular Biology vol. 7 The Humana Press Inc.: Clifton, NJ, 1991.

[bib32] 32Nicklas JA, Brooks EM, Hunter TC, Single R, Branda RF. Development of a quantitative PCR (TaqMan) assay for relative mitochondrial DNA copy number and the common mitochondrial DNA deletion in the rat. Environ Mol Mutagen 2004; 44: 313–320. 15476199 10.1002/em.20050

