# Supplementary material for: Mitochondrial complex II is a source of the reserve respiratory capacity that is regulated by metabolic sensors and promotes cell survival
Source: Cell Death Dis. 2015 Jul 30;6(7):e1835–. doi: 10.1038/cddis.2015.202 (PMC4650745; doi:10.1038/cddis.2015.202)
Supplement: Supplementary Figures [file cddis2015202x1.pdf]

a.

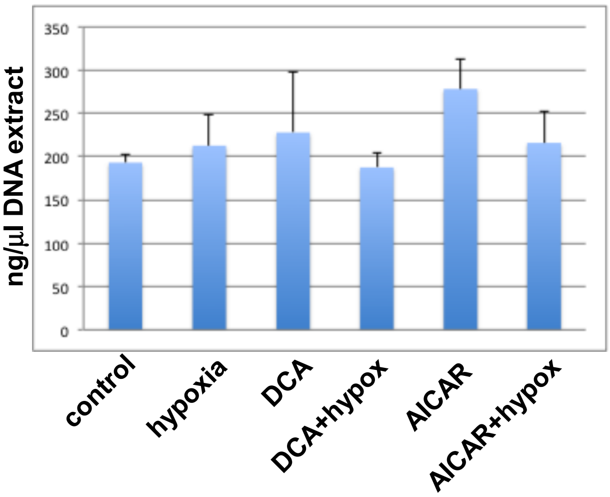

b.

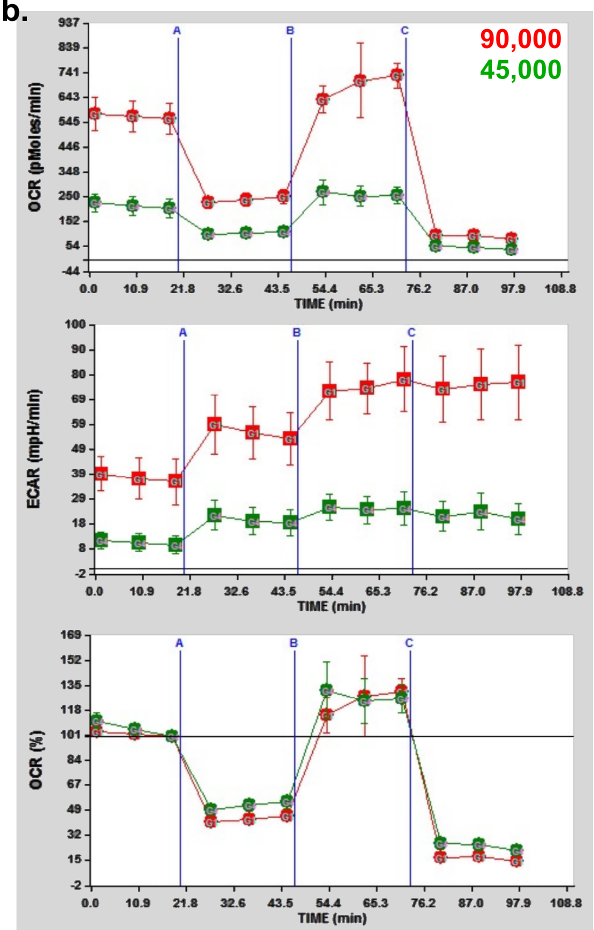

Figure 1S. Effect of 24 hypoxia/reoxygenation on cell number, and the effect of cell number on OCR measurements. a. Cell were subjected to hypoxia in the presence or absence of 1 mM DCA or 500 µM AICAR, as indicated, in the presence of glucose, for 24 h. Following this period the cells were incubated for an additional 24 h in normoxic conditions before DNA was extracted and quantified by qPCR using Gapdh. **b.** Cardiac myocytes were plated at 90,000 and 45,000 cells/per well and OCR (upper panel) and ECAR (middle panel) was measure by the Seahorse analyzer. The lower panel shows the results for OCR from the upper panel plotted as % of basal values.

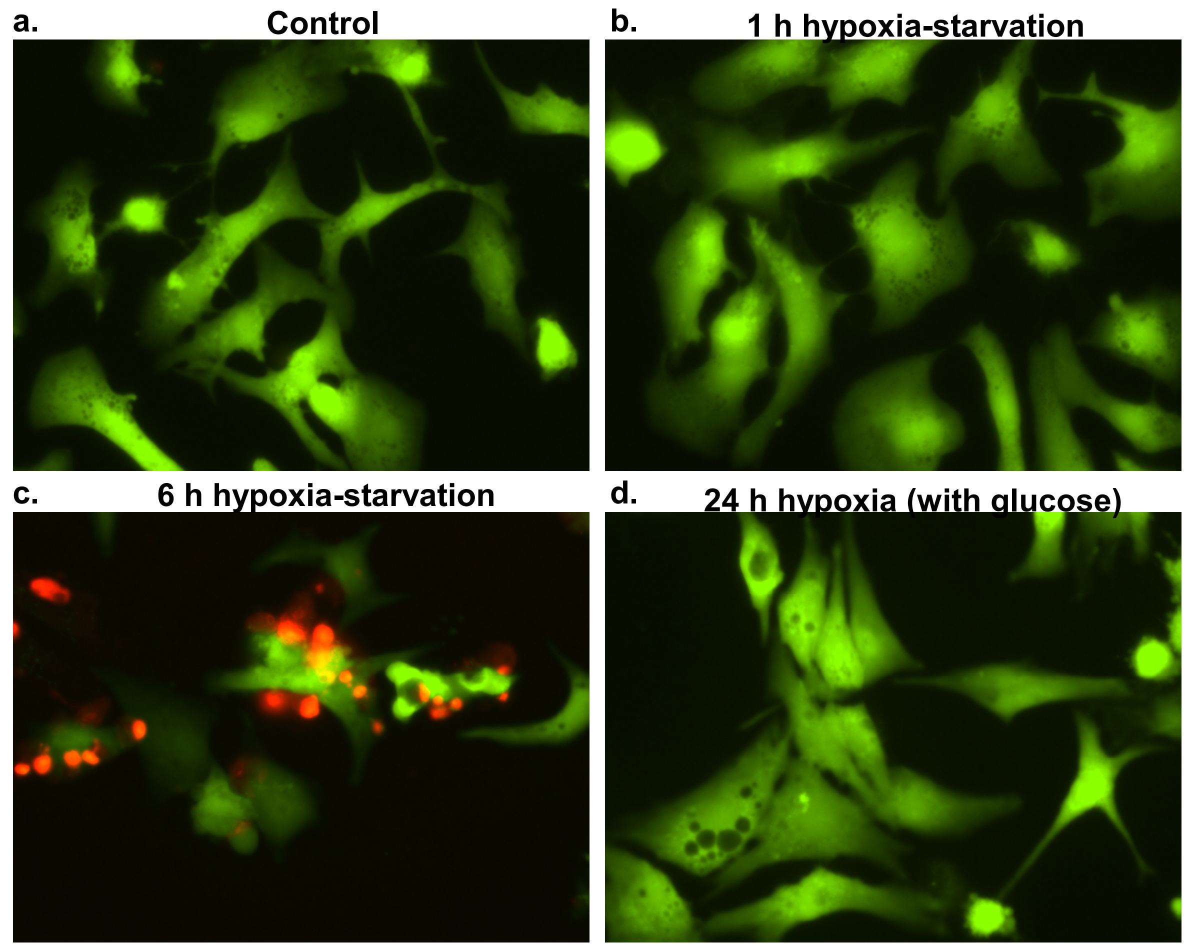

Figure 2S. The effect of hypoxia on cardiac myocyte viability. Cardiac myocytes were **a.** maintained in atmospheric O_2, or were exposed to either **b.** 1 h <1% O_2 with no glucose, **c.** 6 h O_2 with no glucose, or d. 24 h hypoxia in the presence of glucose. Cells were then removed from the hypoxia chamber and were reoxygenated for 30 min-1 h before they were fixed and assyed for live (green) and dead (red nuclei) cells.

Supplementary Figure 2S
